# Craniofacial features of POLR3-related leukodystrophy caused by biallelic variants in *POLR3A*, *POLR3B* and *POLR1C*

**DOI:** 10.1136/jmg-2023-109223

**Published:** 2023-05-16

**Authors:** Amytice Mirchi, Simon-Pierre Guay, Luan T Tran, Nicole I Wolf, Adeline Vanderver, Bernard Brais, Michel Sylvain, Daniela Pohl, Elsa Rossignol, Michael Saito, Sebastien Moutton, Luis González-Gutiérrez-Solana, Isabelle Thiffault, Michael C Kruer, Dolores Gonzales Moron, Marcelo Kauffman, Cyril Goizet, László Sztriha, Emma Glamuzina, Serge B Melançon, Sakkubai Naidu, Jean-Marc Retrouvey, Suzanne Lacombe, Beatriz Bernardino-Cuesta, Isabelle De Bie, Geneviève Bernard

**Affiliations:** 1 Department of Neurology and Neurosurgery, McGill University, Montreal, Quebec, Canada; 2 Department of Pediatrics, McGill University, Montreal, Quebec, Canada; 3 Child Health and Human Development Program, Research Institute of the McGill University Health Centre, Montreal, Quebec, Canada; 4 Department of Human Genetics, McGill University, Montreal, Quebec, Canada; 5 Department of Specialized Medicine, Division of Medical Genetics, McGill University Health Center, Montreal, Quebec, Canada; 6 Department of Child Neurology, Amsterdam Leukodystrophy Center, Emma Children’s Hospital, Amsterdam University Medical Centers, and Amsterdam Neuroscience, Cellular & Molecular Mechanisms, Vrije Universiteit, Amsterdam, Netherlands; 7 Division of Neurology, Children's Hospital of Philadelphia, Philadelphia, Pennsylvania, USA; 8 Department of Neurology, Perelman School of Medicine, University of Pennsylvania, Philadelphia, Pennsylvania, USA; 9 Montreal Neurological Institute, Montreal, Quebec, Canada; 10 Centre Mère Enfant, CHU de Québec, Québec City, Quebec, Canada; 11 Division of Neurology, Children's Hospital of Eastern Ontario, University of Ottawa, Ottawa, Ontario, Canada; 12 Departments of Neurosciences and Pediatrics, CHU-Sainte-Justine, Université de Montréal, Montreal, Quebec, Canada; 13 Department of Pediatrics, University of California Riverside School of Medicine, Riverside Medical Clinic, Riverside, California, USA; 14 Centre Pluridisciplinaire de Diagnostic PréNatal, MSPBordeaux Bagatelle, Talence, France; 15 Sección de Neuropediatría, Hospital Infantil Universitario Niño Jesús, Madrid, España; Grupo Clínico Vinculado al Centro de Investigación Biomédica en Red para Enfermedades Raras (CIBERER) GCV14/ER/6, Hospital Infantil Universitario Nino Jesus, Madrid, Spain; 16 Genomic Medicine Center, Children's Mercy Hospital, Kansas City, Missouri, USA; 17 University of Missouri Kansas City School of Medicine, Kansas City, Missouri, USA; 18 Departments of Child Health, Neurology, and Cellular & Molecular Medicine and Program in Genetics, University of Arizona College of Medicine, Phoenix, Arizona, USA; 19 Programs in Neuroscience and Molecular & Cellular Biology, School of Life Sciences, Arizona State University, Tempe, Arizona, USA; 20 Pediatric Movement Disorders Program, Barrow Neurological Institute, Phoenix Children's Hospital, Phoenix, Arizona, USA; 21 Neurogenetics Unit, Department of Neurology, Hospital JM Ramos Mejia, ADC, Buenos Aires, Argentina; 22 Neurogenetics Unit, Department of Neurology, Hospital JM Ramos Mejia and CONICET-Universidad Austral, Buenos Aires, Argentina; 23 Centre de Référence Neurogénétique, Service de Génétique Médicale, Bordeaux University Hospital, CHU Bordeaux, Bordeaux, France; 24 NRGEN team, INCIA, CNRS UMR 5287, University of Bordeaux, Bordeaux, France; 25 Department of Paediatrics, Albert Szent-Györgyi Medical School, University of Szeged, Szeged, Hungary; 26 Adult and Paediatric National Metabolic Service, Starship Children’s Hospital, Auckland, Te Whatu Ora, New Zealand; 27 Department of Medical Genetics, McGill University Health Centre, Montreal Children’s Hospital, Montreal, Quebec, Canada; 28 Department of Neurogenetics, Kennedy Krieger Institute, Johns Hopkins Medical Institutions, Baltimore, Maryland, USA; 29 Department of Orthodontics, University of Missouri, Kansas City, Missouri, USA; 30 Sección de Neuropediatría, Hospital Infantil Universitario Niño Jesús, Madrid, Spain; 31 Department of Laboratory Medicine, McGill University Health Centre, Montreal, Quebec, Canada

**Keywords:** neurology, genetics, neurodegenerative diseases, genetics, medical, pediatrics

## Abstract

**Background:**

RNA polymerase III-related or 4H leukodystrophy (POLR3-HLD) is an autosomal recessive hypomyelinating leukodystrophy characterized by neurological dysfunction, hypodontia and hypogonadotropic hypogonadism. The disease is caused by biallelic pathogenic variants in *POLR3A*, *POLR3B*, *POLR1C* or *POLR3K*. Craniofacial abnormalities reminiscent of Treacher Collins syndrome have been originally described in patients with POLR3-HLD caused by biallelic pathogenic variants in *POLR1C*. To date, no published studies have appraised in detail the craniofacial features of patients with POLR3-HLD. In this work, the specific craniofacial characteristics of patients with POLR3-HLD associated with biallelic pathogenic variants in *POLR3A*, *POLR3B* and *POLR1C* are described.

**Methods:**

The craniofacial features of 31 patients with POLR3-HLD were evaluated, and potential genotype–phenotype associations were evaluated.

**Results:**

Various craniofacial abnormalities were recognized in this patient cohort, with each individual presenting at least one craniofacial abnormality. The most frequently identified features included a flat midface (61.3%), a smooth philtrum (58.0%) and a pointed chin (51.6%). In patients with *POLR3B* biallelic variants, a thin upper lip was frequent. Craniofacial anomalies involving the forehead were most commonly associated with biallelic variants in *POLR3A* and *POLR3B* while a higher proportion of patients with *POLR1C* biallelic variants demonstrated bitemporal narrowing.

**Conclusion:**

Through this study, we demonstrated that craniofacial abnormalities are common in patients with POLR3-HLD. This report describes in detail the dysmorphic features of POLR3-HLD associated with biallelic variants in *POLR3A*, *POLR3B* and *POLR1C*.

**WHAT IS ALREADY KNOWN ON THIS TOPIC**

- Craniofacial abnormalities in patients harbouring biallelic pathogenic variants in genes encoding different subunits of RNA polymerases including RNA polymerase III have been described only for a specific small subset of phenotypes, that is, Treacher Collins syndrome/POLR1C-related HLD and Wiedemann-Rautenstrauch syndrome. Despite this, description of craniofacial features in individuals with RNA polymerase III-related hypomyelinating leukodystrophy (POLR3-HLD) is currently very limited.

**WHAT THIS STUDY ADDS**

- This is the first study to explore and assess the craniofacial features of a cohort of patients with POLR3-HLD. It is the only study proposing genotype–phenotype correlations based on facial features identified in patients with POLR3-HLD.

**HOW THIS STUDY MIGHT AFFECT RESEARCH, PRACTICE OR POLICY**

- This study is the first to describe the specific phenotypic spectrum of craniofacial anomalies in POLR3-HLD. This detailed account will assist clinicians in diagnosing this condition and will therefore help to provide care directed to this patient population’s specific needs. It will also allow future studies characterizing the underlying pathophysiology of this condition. Indeed, the pathophysiological relationship between biallelic pathogenic variants in a housekeeping gene and specific organ involvement remains to this day unresolved. Characterizing the entire clinical spectrum of this condition will help guide future studies in understanding disease pathogenicity, opening the door for therapy development.

## Introduction

Leukodystrophies are a group of rare heterogenous inherited disorders that affect the cerebral white matter and are typically associated with progressive neurodegeneration.^1^ Although individually rare, they collectively affect 1 in 4733 live births.^2^ The clinical manifestations of this group of disorders can appear at any time from infancy to adulthood and may include developmental delay and/or regression, cerebellar features, gait difficulties, pyramidal and extrapyramidal signs, seizures, cognitive and psychiatric manifestations.^3^

RNA polymerase III-related hypomyelinating leukodystrophy (POLR3-HLD; MIM: 607694, 614381, 616494), one of the most common hypomyelinating leukodystrophies, is an autosomal recessive disorder caused by biallelic pathogenic variants in *POLR3A*, *POLR3B*, *POLR1C or POLR3K*, each encoding subunits of RNA polymerase III.^4–10^ *POLR3A* and *POLR3B* encode the largest subunits that form the catalytic core of RNA polymerase III. *POLR1C* encodes a subunit of both RNA polymerase I and III while *POLR3K* encodes for a different subunit of RNA polymerase III.^4–7 11^ RNA polymerase III is a crucial enzyme responsible for the transcription of small RNAs including transfer RNAs, 5S ribosomal RNA and U6 small nuclear RNA. These are implicated in transcriptional activity regulation, RNA processing, ribosomal assembly and translation necessary for protein synthesis.^12 13^

POLR3-HLD is also known as 4H leukodystrophy in reference to the phenotypic constellation of hypomyelination in addition to hypodontia and hypogonadotropic hypogonadism.^14–17^ Onset of symptoms is typically in early childhood with evidence of motor dysfunction including predominant cerebellar signs in addition to cognitive impairment, abnormal dentition including hypodontia, oligodontia or delayed dentition, endocrinological abnormalities including short stature, delayed or absent puberty and ocular abnormalities, particularly progressive myopia. In addition to the classical hypomyelinating leukodystrophy pattern consisting of mild T2 hyperintensity and variable T1 signal of the white matter compared with grey matter structures, brain MRI typically reveals relative preservation of myelination (i.e., hypointense T2 signal) of specific structures including the dentate nuclei, anterolateral nuclei of the thalami, globi pallidi, pyramidal tracts in the posterior limbs of the internal capsules and optic radiations. In addition, cerebellar atrophy and thinning of the corpus callosum are commonly present.^14 15 18 19^

In recent years, the phenotypic spectrum of POLR3-related disorders has enlarged significantly, including severe neonatal and infantile presentations to late onset mild ones.^20–32^ Reports of craniofacial characteristics of individuals with POLR3-related disorders are scarce and include patients with biallelic pathogenic variants in *POLR1C*,^14^ a gene also associated with Treacher Collins syndrome (TCS), as well as patients with Wiedemann-Rautenstrauch syndrome (WRS) associated with biallelic pathogenic variants in *POLR3A*.^21^ However, to this day, there have been no studies specifically dedicated to exploring the craniofacial features in POLR3-HLD. Here, we further expand the phenotypic description of POLR3-HLD caused by biallelic variants in *POLR3A*, *POLR3B* and *POLR1C* by systematically assessing and characterizing the craniofacial features of 31 identified affected individuals.

## Methods

Thirty-one individuals were included in this single-centre cross-sectional study. The participants were included based on the clinical and radiological features in keeping with a POLR3-HLD diagnosis in addition to biallelic pathogenic or likely pathogenic variants in *POLR3A*, *POLR3B* or *POLR1C* identified by gene panels, exome or genome sequencing using DNA extracted from whole blood according to standard protocols. Interpretation of sequence variants were done as per consensus recommendation of the American College of Medical Genetics and Genomics and the Association for Molecular Pathology.^31^ Only pathogenic and likely pathogenic variants were considered as disease causing. Variants were described based on reference sequence GRCh37 (NM_007055.4 for *POLR3A*, NM_018082.6 for *POLR3B* and NM_203290.4 for *POLR1C*). Compliance with HGVS nomenclature has been verified using VariantValidator. In addition, participants were selected based on availability of photographs of adequate quality for craniofacial analysis. The individuals were recruited at the Montreal Children’s Hospital of the McGill University Health Center between 2012 and 2021.

Facial images including face front and/or profile views of each individual with POLR3-HLD were independently reviewed by two specialists in dysmorphology (SPG and IDB). Both observers were blinded to the genotype. The two physicians performing the dysmorphologic evaluations of patients reviewed and scored all provided pictures independently using ‘Elements of morphology: standard terminology for the head and face’ as a reference.^33^ All evaluations were subsequently revised jointly. There were no instances of significant discordance in scoring and description. Occasional omissions of scoring of some features was the only noted difference. This was resolved through the joint revision of features initially omitted.

Pearson χ^2^ was used to investigate the association between the presence of craniofacial features and the genotype, that is, *POLR3A*, *POLR3B* or *POLR1C* biallelic variants. Only features present in at least 10% of the patients (>3/31) were included for comparison. Identified craniofacial features were also grouped based on their location (forehead, eyes, philtrum, lip and chin). The individual carrying variants in *POLR3A* and *POLR3B* (subject 31) was excluded from the statistical analysis. Results were considered statistically significant when p values were less than 0.05 (two-sided). All statistical analyses were performed with the IBM SPSS Statistics 28 software (release 28.0.0).

## Results

Among the 31 participants, there were 21 males (67.7%) and 10 females (32.3%). All thirty-one participants had a confirmed diagnosis of POLR3-HLD on the basis of their clinical and radiological features in addition to molecularly confirmed presence of likely pathogenic or pathogenic variants in *POLR3A*, *POLR3B* or *POLR1C* (table 1). Variants were present either in the compound heterozygous or homozygous state in each patient. Sixteen participants had biallelic variants in *POLR3A* (51.6%), ten in *POLR3B* (32.2%) and four in *POLR1C* (12.9%). One participant (subject 31) had a combination of a pathogenic and likely pathogenic variant in *POLR3A* in addition to a pathogenic and a deep intronic variant of unknown significance in *POLR3B*. In this participant, we believe that the *POLR3A* variants are disease causing, either solely or in combination with the *POLR3B* variants.

**Table 1.** Description of the pathogenic or likely pathogenic variants identified in our subjects

Subject	Sex	Gene	cDNA variant	Protein	Zygosity	Previous publication(s)
***POLR3A***						
Subject 1	Male	*POLR3A* *POLR3A*	c.1674C>G c.3742_3743insACC	p.F558L p.1248insT	cHET	Bernard *et al* (2010) *Neurogenetics* 43; Bernard *et al* (2011) *Am J Hum Genet* 4; Wolf *et al* (2014) *Neurol* 15; Al Yazidi *et al* (2019) *Mov Disord Clin Pract* 44; Pelletier *et al* (2020) *J Clin Endocr* 45
Subject 2	Male	*POLR3A*	c.2015G>A	p.G672E	HMZ	Bernard *et al* (2010) *Neurogenetics* 43; Bernard *et al* (2011) *Am J Hum Genet* 4; Wolf *et al* (2014) *Neurol* 15; Al Yazidi *et al* (2019) *Mov Disord Clin Pract* 44; Pelletier *et al* (2020) *J Clin Endocr* 45
Subject 3	Male	*POLR3A*	c.2015G>A	p.G672E	HMZ	Bernard *et al* (2010) *Neurogenetics* 43; Bernard *et al* (2011) *Am J Hum Genet* 4; Wolf *et al* (2014) *Neurol* 15; Mirchi *et al* (2018) *Pediatr Neurol* 46; Al Yazidi *et al* (2019) *Mov Disord Clin Pract* 44; Pelletier *et al* (2020) *J Clin Endocr* 45
Subject 4	Female	*POLR3A*	c.2015G>A	p.G672E	HMZ	Bernard *et al* (2011) *Am J Hum Genet* 4; Wolf *et al* (2014) *Neurol* 14; Pelletier *et al* (2020) *J Clin Endocr* 45
Subject 5	Male	*POLR3A* *POLR3A*	c.2015G>A c.3718G>A	p.G672E p.G1240S	cHET	Wolf *et al* (2014) *Neurol* 14; Mirchi *et al* (2018) *Pediatr Neurol* 46; Al Yazidi *et al* (2019) *Mov Disord Clin Pract* 44; Pelletier *et al* (2020) *J Clin Endocr* 45
Subject 6	Male	*POLR3A* *POLR3A*	c.1674C>G c.2015G>A	p.F558L p.G672E	cHET	Wolf *et al* (2014) *Neurol* 15; Mirchi *et al* (2018) *Pediatr Neurol* 46; Al Yazidi *et al* (2019) *Mov Disord Clin Pract* 44; Pelletier *et al* (2020) *J Clin Endocr* 45
Subject 7	Male	*POLR3A* *POLR3A*	c.3583del c.1771–7C>G	p.D1195Ifs*47 p.E548_Y637del/p.P591Mfs*9	cHET	Perrier *et al* (2020) *Neurol-Genet* 20
Subject 8	Male	*POLR3A* *POLR3A*	c.1771–6C>G c.3205C>T	p.P591Mfs*9 p.R1069W	cHET	La Piana *et al* (2016) *Neurol* 25; Pelletier *et al* (2020) *J Clin Endocr* 45
Subject 9	Female	*POLR3A* *POLR3A*	c.3014G>A c.3781G>A	p.R1005H p.E1261K	cHET	Wolf *et al* (2014) *Neurol* 15; Mirchi *et al* (2018) *Pediatr Neurol* 46; Cordoba *et al* (2018) *PLoS One* 47; Al Yazidi *et al* (2019) *Mov Disord Clin Pract* 44; Pelletier *et al* (2020) *J Clin Endocr* 45
Subject 10	Male	*POLR3A* *POLR3A*	c.1771–6C>G c.2819_2820del	p.P591Mfs*9 p.L940Qfs*17	cHET	N/A
Subject 11	Male	*POLR3A* *POLR3A*	c.1771–7C>G c.3387C>A	p.E548_Y637del/p.P591Mfs*9 p.L1129=	cHET	Harting *et al* (2020) *Neurogenetics* 27
Subject 12	Female	*POLR3A* *POLR3A*	c.1369G>A c.3242+2A>G	p.G457R –	cHET	Mirchi *et al* (2018) *Pediatr Neurol* 46; Al Yazidi *et al* (2019) *Mov Disord Clin Pract* 44; Pelletier *et al* (2020) *J Clin Endocr* 45
Subject 13	Female	*POLR3A* *POLR3A*	c.2554A>G c.2617–1G>A	p.M852V p.R873Afs*878	cHET	Timmons *et al* (2006) *Neurol* 16; Bernard *et al* (2011) *Am J Hum Genet* 4; Wolf *et al* (2014) *Neurol* 15; Al Yazidi *et al* (2019) *Mov Disord Clin Pract* 44; Pelletier *et al* (2020) *J Clin Endocr* 45
Subject 14	Male	*POLR3A* *POLR3A*	c.1186G>T c.2015G>A	p.V396L p.G672E	cHET	Wolf *et al* (2014) *Neurol* 15; Mirchi *et al* (2018) *Pediatr Neurol* 46; Al Yazidi *et al* (2019) *Mov Disord Clin Pract* 44; Pelletier *et al* (2020) *J Clin Endocr* 45
Subject 15	Male	*POLR3A*	c.1909+18G>A	p.Y637Cfs*14	HMZ	Al Yazidi *et al* (2019) *Mov Disord Clin Pract* 44; Pelletier *et al* (2020) *J Clin Endocr* 45
Subject 16	Male	*POLR3A* *POLR3A*	c.1051C>T c.1771–7C>G	p.R351* p.E548_Y637del/p.P591Mfs*9	cHET	Perrier *et al* (2020) *Neurol-Genet* 20
***POLR3B***						
Subject 17	Female	*POLR3B*	c.1324C>T c.1568T>A	p.R442C p.V523E	cHET	Wolf *et al* (2014) *Neurol* 15; Daoud *et al* (2013) *J Med Genet* 9; Al Yazidi *et al* (2019) *Mov Disord Clin Pract* 44; Pelletier *et al* (2020) *J Clin Endocr* 45
Subject 18	Female	*POLR3B*	c.1568T>A	p.V523E	HMZ	Wolf *et al* (2014) *Neurol* 15; Al Yazidi *et al* (2019) *Mov Disord Clin Pract* 44; Perrier *et al* (2020) *Neurol-Genet* 20; DeGasperis *et al* (2020) *Neurol-Genet* 48; Pelletier *et al* (2020) *J Clin Endocr* 45
Subject 19	Male	*POLR3B*	c.1568T>A	p.V523E	HMZ	Wolf *et al* (2014) *Neurol* 15; Perrier *et al* (2020) *Neurol-Genet* 20; DeGasperis *et al* (2020) *Neurol-Genet* 48; Pelletier *et al* (2020) *J Clin Endocr* 45
Subject 20	Male	*POLR3B*	c.312G>T c.2570+1G>A	p.L104F p.G818fs	cHET	Wolf *et al* (2014) *Neurol* 15; Mirchi *et al* (2018) *Pediatr Neurol* 46
Subject 21	Male	*POLR3B*	c.1568T>A c.1947_1951del	p.V523E p.N650Lfs*46	cHET	Mirchi *et al* (2018) *Pediatr Neurol* 46; Pelletier *et al* (2020) *J Clin Endocr* 45
Subject 22	Male	*POLR3B*	c.496+3A>G	–	HMZ	N/A
Subject 23	Female	*POLR3B*	c.1568T>A c.2740G>A	p.V523E p.E914K	cHET	N/A
Subject 24	Male	*POLR3B*	c.1999G>A c.2084–6A>G	p.V667M p.G695Vfs*5	cHET	N/A
Subject 25	Male	*POLR3B*	c.1999G>A c.2084–6A>G	p.V667M p.G695Vfs*5	cHET	N/A
Subject 26	Female	*POLR3B*	c.1568T>A c.2818–2A>T	p.V523E –	cHET	Mirchi *et al* (2018) *Pediatr Neurol* 46
***POLR1C***						
Subject 27	Male	*POLR1C*	c.88C>T c.615del	p.P30S p.Q206Kfs*48	cHET	Gauquelin *et al* (2019) *Neurol-Genet* 14
Subject 28	Female	*POLR1C*	c.699C>G c.883_885del	p.Y233* p.K295del	cHET	Gauquelin *et al* (2019) *Neurol-Genet* 14
Subject 29	Female	*POLR1C*	c.77C>T c.326G>A	p.T26I p.R109H	cHET	Thiffault *et al* (2015) *Nat Commun* 6; Gauquelin *et al* (2019) *Neurol-Genet* 14
Subject 30	Male	*POLR1C*	c.221A>G	p.N74S	HMZ	Thiffault *et al* (2015) *Nat Commun* 6; Gauquelin *et al* (2019) *Neurol-Genet* 14
***POLR3A (±POLR3B)***						
Subject 31*	Male	*POLR3A* *POLR3A* *POLR3B* *POLR3B*	c.2434G>A deletion of exon 6-8 c.1006G>A c.72+294C>A†	p.G812S – p.A336T –	cHET cHET	Mirchi *et al* (2018) *Pediatr Neurol* 46; Pelletier *et al* (2020) *J Clin Endocr* 45

DNA sequence variants were described based on referring sequence NM_007055.4 for *POLR3A*, NM_018082.6 for *POLR3B* and NM_203290.4 for *POLR1C* (GRCh37).

*In this patient, the biallelic *POLR3A* variants are disease causing, either solely or in combination with the *POLR3B* variants

†This variant is of unknown significance

cHET, compound heterozygous; HMZ, homozygous.

**Figure 1:**
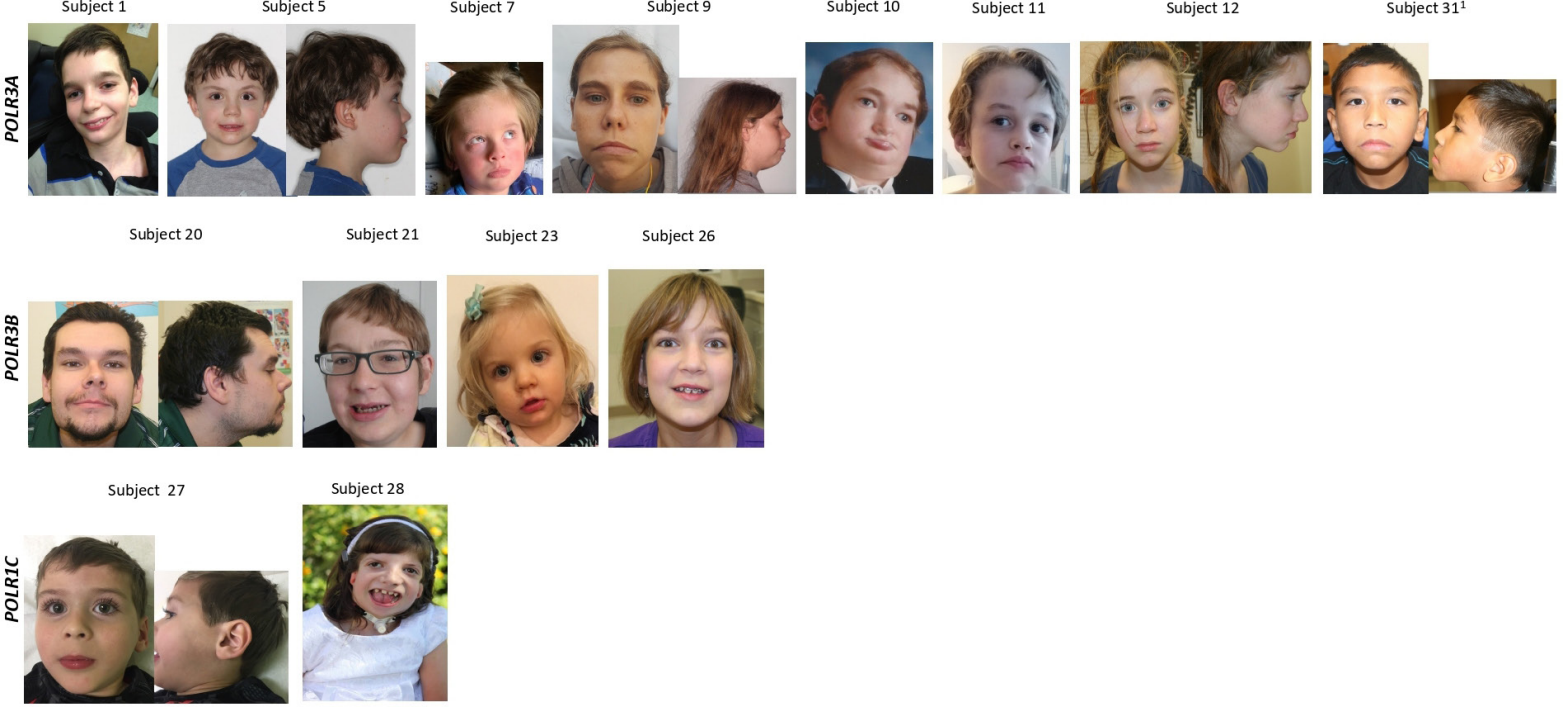
Craniofacial characteristics in patients with POLR3-HLD by genotype. Selection of representative pictures of our patient cohort are shown. Anomaly of the lower face including a flat midface (subjects 5, 7, 9, 12, 20, 23, 27, 31), smooth philtrum (subjects 1, 9, 12, 21, 26, 27, 31) and pointed chin (subjects 1, 5, 11, 12, 21, 26, 27, 31) were among the most common craniofacial features in our cohort of patients. ^1^This patient also has a variant of unknown significance and a pathogenic variant in *POLR3B.*

**Figure 2:**
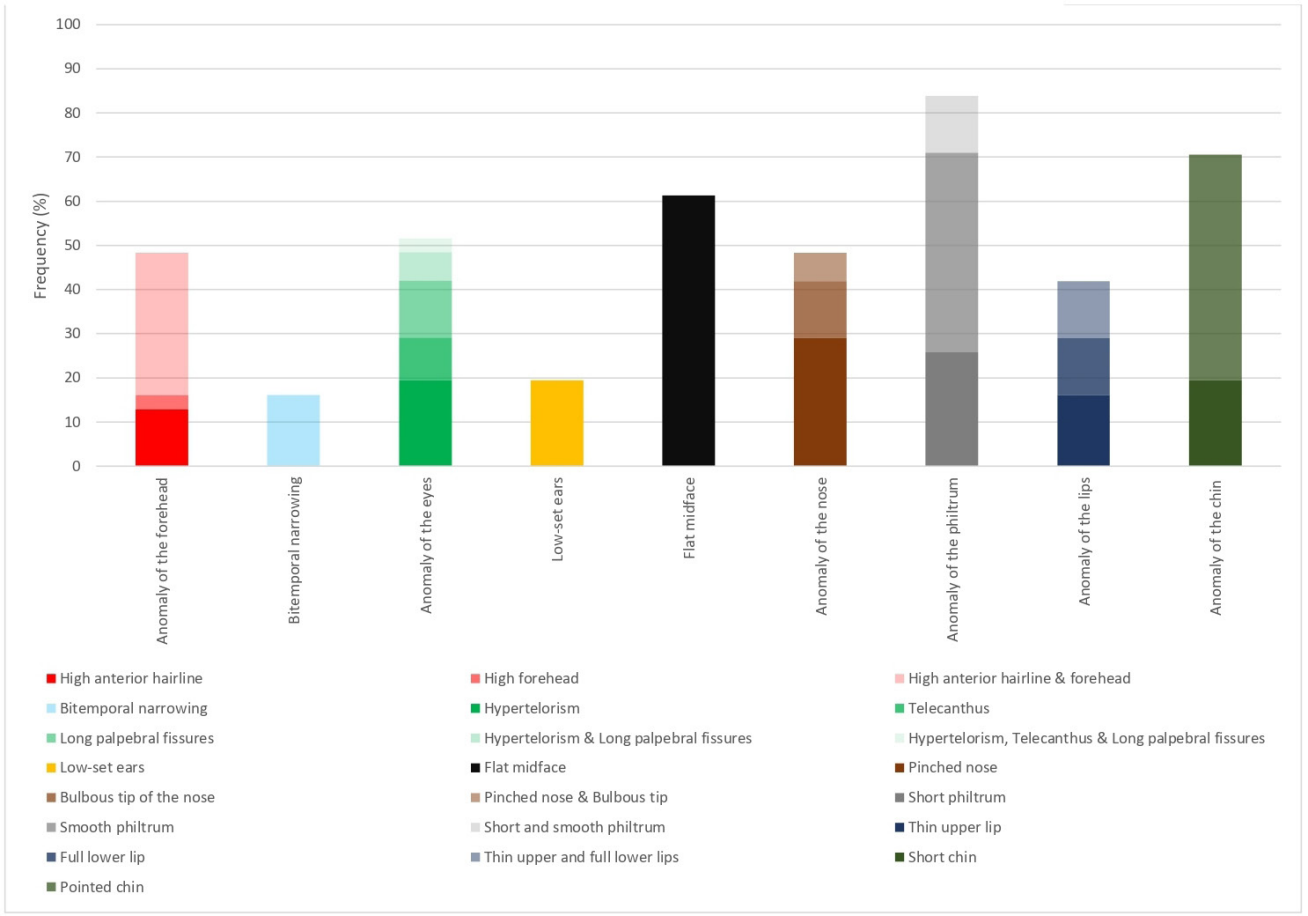
Frequency of the craniofacial features described in our cohort of patients with POLR3-HLD.

All individuals presented at least one craniofacial abnormality (figure 1 and online supplemental file 1). Although some of these could be familial, a subset of craniofacial abnormalities was described in more than 50% of the individuals. In total, 16 craniofacial abnormalities were recognized in at least 10% of the individuals, including a high anterior hairline, high forehead, bitemporal narrowing, hypertelorism, telecanthus, long palpebral fissures, low-set ears, flat midface, pinched nose, bulbous tip of the nose, short and/or smooth philtrum, thin upper lip, full lower lip, short chin and pointed chin. Our analysis revealed that more than half of the subjects in our cohort have craniofacial abnormalities involving the eyes, the midface, the philtrum or the chin. A flat midface (61.3%; 19/31), smooth philtrum (58.0%; 18/31) and pointed chin (51.6%; 16/31) were the most common craniofacial features observed (figure 2). Moreover, 83.9% (26/31) of subjects had an anomaly of the philtrum with either a short and/or smooth philtrum. Seventy-one per cent (22/31) of patients had an anomaly of the chin consisting of a short and/or pointed chin. An anomaly of the eyes was seen in 51.6% (16/31) of subjects with hypertelorism, telecanthus and/or long palpebral fissures. Interestingly, subject 28, previously published,^14^ who has biallelic variants in *POLR1C*, whose photograph is shown in figure 1, displayed some craniofacial features typically observed in TCS including bitemporal narrowing, downslanting palpebral fissures and abnormalities of the external ears.

10.1136/jmg-2023-109223.supp1 Supplementary data

As shown in table 2, comparisons of the craniofacial abnormalities based on underlying genotype revealed some distinctive features between the three groups of patients. More specifically, a statistically significant difference was identified between genotypes and the presence of a thin upper lip. Patients with biallelic variants in *POLR3B* were found to most frequently display a thin upper lip as opposed to patients with *POLR3A* and *POLR1C* biallelic variants (p=0.036). *POLR3B* patients were identified more frequently as presenting a thin upper lip compared with *POLR3A* patients (p=0.011). Craniofacial abnormalities involving the forehead characterized as a high anterior hairline and/or a high forehead were found to be most common in individuals with *POLR3A* (50.0%; 8/16) and *POLR3B* (60%; 6/10) pathogenic variants as opposed to *POLR1C*, with none of our four *POLR1C* patients being described as having an anomaly of the forehead. There was a statistically significant difference between the *POLR3B* and *POLR1C* groups with a p value of 0.040 when evaluating for the presence of an anomaly of the forehead. On the other hand, bitemporal narrowing was identified most commonly in patients with *POLR1C* variants (50.0%; 2/4) as opposed to patients with *POLR3A* variants (18.8%; 3/16). Bitemporal narrowing was absent in all our patients with *POLR3B* variants. When comparing the groups of patients with *POLR3B* and *POLR1C* variants, there was a statistically significant difference supporting that the presence of bitemporal narrowing is most commonly seen in *POLR1C* patients (p=0.016).

**Table 2:** Craniofacial features of POLR3-HLD patients according to genotype

Craniofacial features	*POLR3A* (n=16)	*POLR3B* (n=10)	*POLR1C* (n=4)	p-value	p -value (3A vs 3B)	p -value (3A vs 1C)	p -value (3B vs 1C)
High anterior hairline	8 (50.0)	5 (50.0)	0	0.171	1	*0.068*	*0.078*
High forehead	6 (37.5)	4 (40.0)	0	0.313	0.899	0.143	0.134
Anomaly of the forehead*	8 (50.0)	6 (60.0)	0	0.117	0.619	*0.068*	**0.040**
Bitemporal narrowing	3 (18.8)	0	2 (50.0)	*0.072*	0.145	0.197	**0.016**
Hypertelorism	6 (37.5)	2 (20.0)	1 (25.0)	0.621	0.347	0.639	0.837
Telecanthus	1 (6.3)	2 (20.0)	1 (25.0)	0.461	0.286	0.264	0.837
Long palpebral fissures	3 (18.8)	4 (40.0)	0	0.228	0.235	0.348	0.134
Anomaly of the eyes†	7 (43.8)	7 (70.0)	2 (50.0)	0.422	0.191	0.822	0.480
Low-set ears	2 (12.5)	2 (20.0)	1 (25.0)	0.787	0.606	0.531	0.837
Flat midface	10 (62.5)	6 (60.0)	2 (50.0)	0.901	0.899	0.648	0.733
Pinched nose	6 (37.5)	4 (40.0)	1 (25.0)	0.866	0.899	0.639	0.597
Bulbous tip of the nose	3 (18.8)	1 (10.0)	1 (25.0)	0.752	0.547	0.780	0.469
Anomaly of the nose‡	8 (50.0)	4 (40.0)	2 (50.0)	0.875	0.619	1	0.733
Short philtrum	7 (43.8)	3 (30.0)	2 (50.0)	0.713	0.483	0.822	0.480
Smooth philtrum	7 (43.8)	8 (80.0)	2 (50.0)	0.185	*0.069*	0.822	0.262
Anomaly of the philtrum§	13 (81.3)	8 (80.0)	4 (100)	0.628	0.937	0.348	0.334
Thin upper lip	2 (12.5)	6 (60.0)	1 (25.0)	**0.036**	**0.011**	0.531	0.237
Full lower lip	3 (18.8)	4 (40.0)	1 (25.0)	0.490	0.235	0.780	0.597
Anomaly of the lips¶	4 (25.0)	7 (70.0)	2 (50.0)	*0.076*	**0.024**	0.329	0.480
Short chin	3 (18.8)	3 (30.0)	0	0.440	0.508	0.348	0.217
Pointed chin	9 (56.3)	3 (30.0)	3 (75.0)	0.241	0.191	0.494	0.124
Anomaly of the chin**	12 (75.0)	6 (60.0)	3 (75.0)	0.700	0.420	1	0.597

Pearson χ^2^ was used to investigate the association between the presence of craniofacial features and the genotype.

P-values are reported for the 3-group comparison (1st column) as well as 2-group comparisons (2nd, 3rd and 4th columns). A p-value below 0.05 suggest a statistical difference for the craniofacial feature prevalence between the groups and are shown in bold. P-values below 0.10 are shown in italic. Only features present in at least 10% of the sample (>3/31) were included for comparison. The individual carrying pathogenic variants in *POLR3A* and 1 pathogenic variant and 1 variant of unknown significance in *POLR3B* (subject 31) was excluded from the statistical analysis (n=30). Identified craniofacial features were also grouped based on their location.

*Anomaly of the forehead included a high anterior hairline or a high forehead.

†Anomaly of the eyes included hypertelorism, telecanthus or long palpebral fissures.

‡Anomaly of the nose included a pinched nose or a bulbous tip of the nose.

§Anomaly of the philtrum included a short or smooth philtrum.

¶Anomaly of the lips included a thin upper or a full lower lip.

**Anomaly of the chin included a short or pointed chin.

## Discussion

Our study illustrates the various craniofacial features present in patients with POLR3-HLD caused by biallelic variants in *POLR3A*, *POLR3B* and *POLR1C*. Anomalies of the lower face including a flat midface, smooth philtrum and pointed chin were among the most common craniofacial features in our cohort of patients. In addition, genotype–phenotype correlations enabled the identification of differences between the craniofacial features and underlying genotype of patients. Presence of a thin upper lip was most frequently associated with *POLR3B* biallelic variants while patients with *POLR3A* variants were most commonly found to have forehead abnormalities. In addition, bitemporal narrowing was associated with underlying *POLR1C* biallelic variants. The gene-specific dysmorphic features described in this study are additional clues that could help clinicians suspect POLR3-HLD in patients presenting with a hypomyelinating leukodystrophy.

Specific craniofacial characteristics have previously been associated with biallelic variants in various genes encoding four RNA polymerase III subunits. WRS is a neonatal progeroid disorder characterized by premature ageing and associated with intrauterine growth restriction, postnatal growth failure, short stature, lipodystrophy, hypotonia and intellectual disability.^21 23 34^ A previous study in 2018 identified specific combinations of biallelic *POLR3A* variants associated with WRS. It was hypothesized that the specific combinations of compound heterozygous variants in this gene correlate with the WRS disease phenotype.^14 20^ Individuals with WRS typically have a characteristic facial appearance with a triangular facies, sparse scalp hair, an enlarged fontanelle, prominent scalp veins, a pointed chin, a convex or pinched nose, low-set eyes, a small mouth and dental abnormalities reminiscent of what can be seen in patients with POLR3-HLD including presence of natal teeth or hypodontia.^21 23 34^ In 2021, report of pathogenic compound heterozygous variants in *POLR3B* in a patient with WRS led to further expansion of the genotypic spectrum of this condition.^23^ A prior study has also identified a nonsense variant in *POLR3GL*, a gene encoding another subunit of RNA polymerase III, as being associated with WRS.^22^

TCS is a disorder presenting with specific craniofacial features caused by defects of embryogenesis of the first and second brachial arches, most often transmitted as an autosomal dominant condition. TCS is characterized by downslanting palpebral fissures, facial bone hypoplasia, micrognathia and external ear anomalies including microtia in addition to conductive hearing loss.^35–37^ Some individuals with TCS may also have a cleft palate or choanal atresia.^37^ Although TCS is most frequently attributed to heterozygous pathogenic variants in *TCOF1*, rarer forms of this condition result from heterozygous pathogenic variant in *POLR1B* or *POLR1D*, or biallelic pathogenic variants in *POLR1C* or *POLR1D*.^36 37^ In 2019, Gauquelin and colleagues characterized, in a multicentre study, the clinical spectrum of 23 patients with POLR3-HLD caused by biallelic pathogenic variants in *POLR1C*. In their cohort of patients, one had craniofacial features compatible with TCS including downslanting palpebral fissures, strabismus, bitemporal narrowing, external ear anomaly, cleft palate and micrognathia corresponding to subject 28 in our cohort. Four patients had more subtle craniofacial anomalies with mild mandibular hypoplasia and one patient had laryngomalacia. Their results illustrated that POLR1C-related HLD can be associated with craniofacial features reminiscent of TCS.^14^ Prior *in vitro* functional studies have demonstrated that mutations in *POLR1C* associated with POLR3-HLD prevent assembly and targeting of RNA polymerase III to the nucleus but not RNA polymerase I. In contrast, a TCS-causing mutation, p.Arg279Gln, was shown not to affect assembly of either polymerases but rather impaired targeting of RNA polymerase I to the nucleolus.^6^ This study was the first illustrating the concept that mutations in *POLR1C* coding for a subunit common to RNA polymerase I and RNA polymerase III can lead to different effects on these two protein complexes and therefore result in different or combined phenotypes. This work provided a potential pathophysiologic mechanism underlying the phenotypic heterogeneity seen with mutations in this gene.^6^ However, in a later cohort of patients described by Gauquelin and colleagues in 2019, two participants were carrying the pathogenic variant p.Arg279Gln previously associated with TCS, yet none showed abnormal craniofacial development suggesting that the underlying pathophysiological mechanism is likely even more complex and raising the question of implications of genetic modifiers influencing the pathophysiology of POLR1C-related disorders.^14^

Development of craniofacial structures is a complex process occurring in an orderly fashion throughout embryonic and fetal stages. Craniofacial growth occurs due to a relatively rapid and orderly composition of mesodermal and cranial neural crest cells involved in the first and second branchial arch formation.^38^ Interestingly, generation of insufficient neural crest cells is a known mechanism leading to general craniofacial anomalies described in *TCOF1*, *POLR1C* and *POLR1D*-related TCS.^39 40^ Indeed, haploinsufficiency of *Tcof1* in mice and *Polr1c* or *Polr1d* in zebrafish results in deficient ribosome biogenesis, which is incapable of meeting the proliferative needs of the neuroepithelium and leads to a high degree of neuroepithelial apoptosis.^40 41^ Interestingly, the craniofacial features described here for individuals with biallelic pathogenic or likely pathogenic variants in *POLR3A* and *POLR3B* could also be potentially explained by perturbation of the neural crest cells. We hypothesize that the decrease in POLR3A or POLR3B impairs RNA polymerase III biogenesis leading to dysregulation of the expression of certain RNA polymerase III targets and thereby perturbating cytoplasmic protein synthesis essential for neural crest cell development.^4 42^ This reduced RNA and protein production may alter the proliferation of neuroepithelium and similarly lead to neuroepithelial apoptosis as seen in *Tcof1*-haploinsufficient cells in mice or *Polr1c*/*Polr1d*-haploinsufficient cells in zebrafish. However, further studies are required to confirm this hypothesis.

As illustrated with this study, craniofacial abnormalities are common among individuals with POLR3-HLD. In this cohort of patients with pathogenic or likely pathogenic biallelic variants in *POLR3A*, *POLR3B* and *POLR1C,* each patient presented at least one craniofacial abnormality. This work further expands the phenotypic spectrum of POLR3-HLD. We present a novel group of craniofacial features associated with POLR3-HLD from what has been previously described in the literature, with the exception of the TCS craniofacial features previously described in a study by Gauquelin and colleagues.^14^ One limitation of this study is that the description of dysmorphic features was limited by the number of pictures available for some patients. Another limitation is the small sample size. Nevertheless, sample size is quite large considering that POLR3-HLD is a rare condition. Moreover, parental pictures were not available to determine if some of the facial features could be familial in nature. However, the independent analysis of pictures by two physicians experienced in dysmorphology clearly established the presence of craniofacial abnormalities mainly affecting the lower face associated with pathogenic variants in genes encoding RNA polymerase III subunits.

In conclusion, with this addition to the detailed characterization of the disease phenotype, we hope for early recognition and diagnosis of individuals with POLR3-HLD, an important task for clinicians in an era where clinical trial development and advancement in gene therapy for rare neurodegenerative disorders has been booming. Detailed phenotyping of the condition also allows for further genotype–phenotype correlations and contribute to the advancement in understanding the pathophysiology underlying POLR3-HLD.

## Data Availability

All data relevant to the study are included in the article or uploaded as supplementary information.
