# Supplementary material for: Craniofacial features of POLR3-related leukodystrophy caused by biallelic variants in POLR3A, POLR3B and POLR1C
Source: J Med Genet. 2023 May 16;60(10):1026–34. doi: 10.1136/jmg-2023-109223 (PMC10579516; doi:10.1136/jmg-2023-109223)
Supplement: Supplementary data [file jmg-2023-109223supp001.pdf]

Supplemental material. Description of the craniofacial features of the 31 POLR3-HDL subjects in this study's cohort

Subject	Gene	High anterior hairline	High forehead	Bitemporal narrowing	Hypertelorism	Telecanthus	Long palpebral fissures	Low-set ears	Flat midface	Pinched nose	Bulbous tip of the nose	Short philtrum	Smooth philtrum	Thin upper lip	Full lower lip	Short chin	Pointed chin	Other dysmorphic features (observed in < 10%)
1	*POLR3A*	+			+	+	+					+	+				+	Large mouth, bilateral syndactyly 2-3 toes
2	*POLR3A*			+	+		+		+	+				+				
3	*POLR3A*								+	+		+					+	
4	*POLR3A*				+			+									+	Chin dimple
5	*POLR3A*						+	+	+		+	+					+	
6	*POLR3A*	+	+						+	+	+					+		Sparing of lateral eyebrows
7	*POLR3A*	+	+		+				+			+				+		Upturned nose
8	*POLR3A*								+				+				+	
9	*POLR3A*	+	+						+				+		+			Deviating nasal root, retrognathia
10	*POLR3A*			+	+							+				+		Short neck
11	*POLR3A*	+	+							+		+					+	Triangular face
12	*POLR3A*								+		+		+				+	
13	*POLR3A*	+								+			+				+	Ptosis, bifid tip of the nose
14	*POLR3A*	+	+	+					+				+					Ptosis, downslanting palpebral fissure, mildly overfoled helixes, prognatism
15	*POLR3A*								+	+		+		+	+			Hypotelorism, broad nasal tip, low-hanging columella
16	*POLR3A*	+	+		+								+		+		+	Sparce medial eyebrows, broad nasal bridge
17	*POLR3B*	+					+		+			+	+	+	+	+		Broad and flat nasal bridge
18	*POLR3B*									+			+					Depressed nasal bridge, retrognathia
19	*POLR3B*								+	+	+		+	+		+		
20	*POLR3B*							+	+	+								Deviating nasal root
21	*POLR3B*	+				+						+	+	+	+		+	Large ears
22	*POLR3B*				+		+		+				+	+	+		+	Short nose
23	*POLR3B*	+	+			+			+						+	+		Inferior epicanthus
24	*POLR3B*	+	+				+		+				+	+				Hockey-stick palmar creases
25	*POLR3B*	+	+				+			+			+	+				Hockey-stick palmar creases
26	*POLR3B*		+		+			+				+	+				+	Copped ears, low hanging columella
27	*POLR1C*			+		+		+	+				+		+		+	Long eyelashes, long philtrum, inferior epicanthus
28	*POLR1C*			+	+							+						Downslanted palpebral fissure, large mouth, short neck, abnormal external ears
29	*POLR1C*										+	+		+			+	Peri-orbital fullness, low-hanging columella
30	*POLR1C*								+	+			+				+	Broad nasal bridge, retrognathia
31	*POLR3A (+/- POLR3B)*	+	+					+	+		+		+				+	Epicanthal fold
		14/31 (45.2%)	11/31 (35.4%)	5/31 (16.1%)	9/31 (29.0%)	4/31 (12.9%)	7/31 (22.6%)	6/31 (19.4%)	19/31 (61.3%)	11/31 (35.5%)	6/31 (22.6%)	12/31 (38.7%)	18/31 (58.0%)	9/31 (29.0%)	8/31 (25.8%)	6/31 (19.4%)	16/31 (51.6%)	
		15/31 with an anomaly of the forehead (48.4%)			16/31 with an anomaly of the eyes (51.6%)					15/31 with an anomaly of the nose (48.4%)		26/31 with an anomaly of the philtrum (83.9%)		13/31 with an anomaly of the lip (41.9%)		22/31 with an anomaly of the chin (71.0%)		
